# Correction: Alathari et al. A Multiplexed, Tiled PCR Method for Rapid Whole-Genome Sequencing of Infectious Spleen and Kidney Necrosis Virus (ISKNV) in Tilapia. *Viruses* 2023, *15*, 965

**DOI:** 10.3390/v15071476

**Published:** 2023-06-29

**Authors:** Shayma Alathari, Dominique L. Chaput, Luis M. Bolaños, Andrew Joseph, Victoria L. N. Jackson, David Verner-Jeffreys, Richard Paley, Charles R. Tyler, Ben Temperton

**Affiliations:** 1 Department of Biosciences, University of Exeter, Stocker Road, Exeter EX4 4QD, UK; 2 Centre for Environment, Fisheries and Aquaculture Science (Cefas), The Nothe, Barrack Road, Weymouth DT4 8UB, UK; 3 Sustainable Aquaculture Futures Centre, University of Exeter, Exeter EX4 4QD, UK

In the original publication [1], there was a mistake in Figure 1 and Figure 2 as published. 

In Figure 1, there is a loss of colour in most of the dots representing the farms on the map. The corrected Figure 1 appears below.

In Figure 2, there is a loss of all dots. The corrected Figure 2 appears below. 

The authors state that the scientific conclusions are unaffected. This correction was approved by the Academic Editor. The original publication has also been updated.

## Figures and Tables

**Figure 1:**
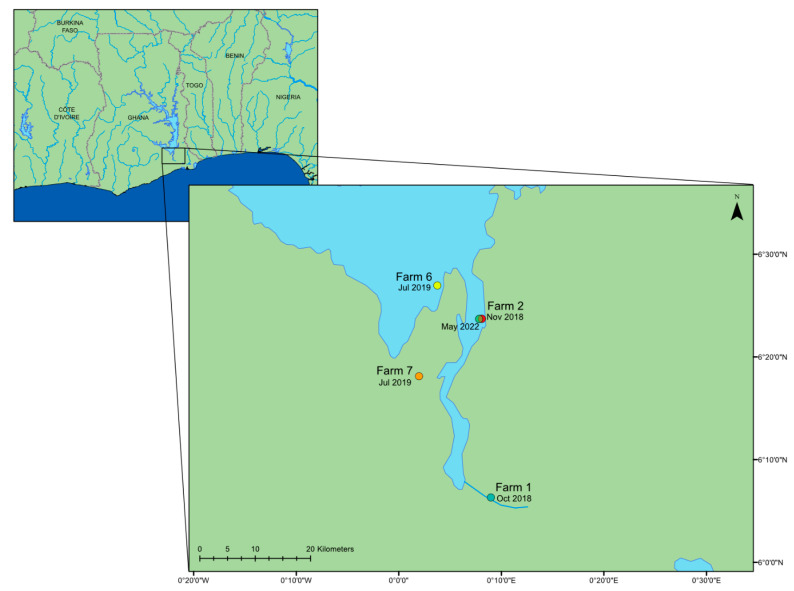
A map of the lower region of Lake Volta in Ghana, West Africa, showing the date and location of the farms where the outbreaks of mortality occurred; locations retrieved from [11]. This map was constructed using ArcGIS (GIS software). Version 10.0. Redlands, CA, USA: Environmental Systems Research Institute, Inc., 2010.

**Figure 2:**
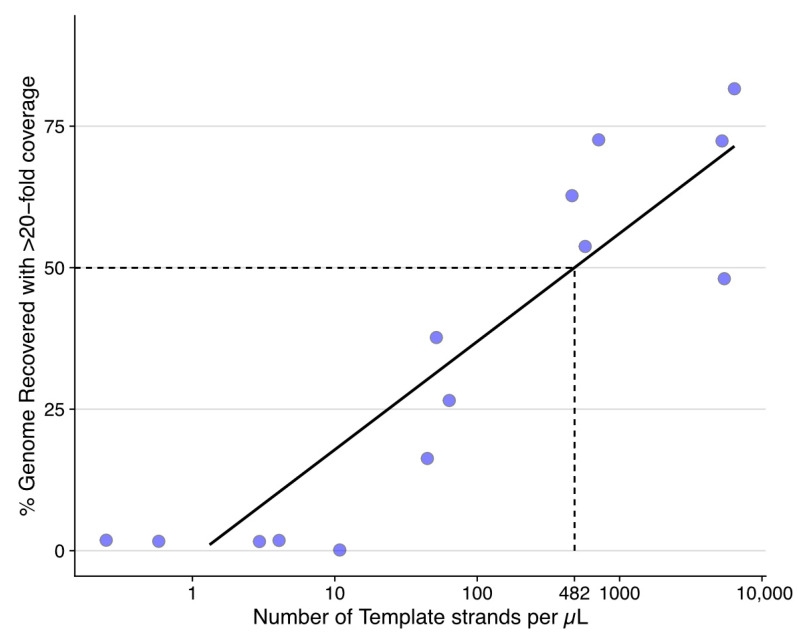
Successful recovery of >50% of the ISKNV genome required 482 template strands per µL (2410 viral templates per 5 µL sequencing reaction), with a minimum of 0.2 copies per µL to recover >0% of the genome with at least 20-fold coverage for error correction. Number of viral templates was measured using ddPCR from a serially diluted ISKNV template, which was subsequently sequenced and processed as described in the text.
