# Supplementary material for: A Multiplexed, Tiled PCR Method for Rapid Whole-Genome Sequencing of Infectious Spleen and Kidney Necrosis Virus (ISKNV) in Tilapia
Source: Viruses. 2023 Apr 14;15(4):965. doi: 10.3390/v15040965 (PMC10145788; doi:10.3390/v15040965)
Supplement: Supplementary file 1 [file viruses-15-00965-s001.zip › viruses-2291360-supplementary.pdf]

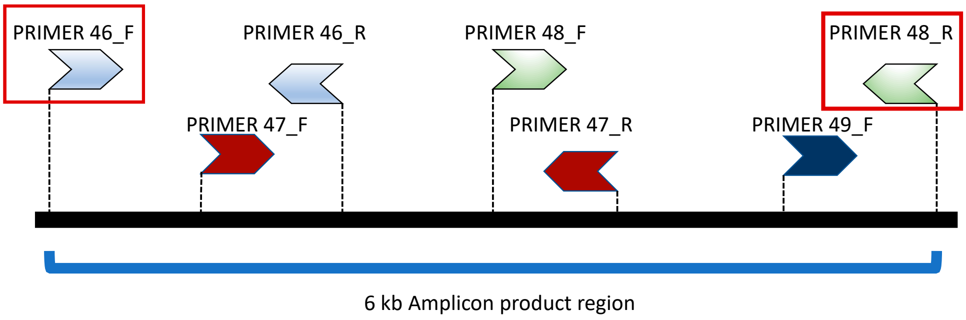

Figure S1. A schematic diagram for the 6kb amplicon product targeting the primer 47 region. Product selected using the 46f and 48r primers (circled in red) from the generated primer sets for ISKNV.

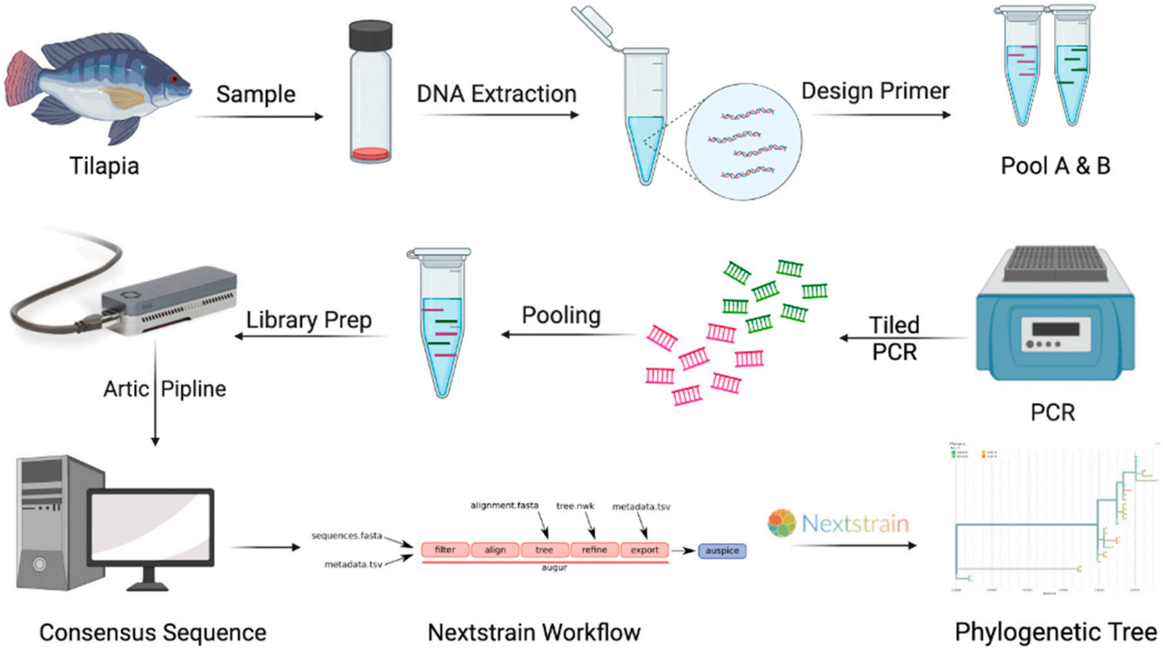

Figure S2. An illustration of processing tissue samples collected from infected fish, across the ISKNV outbreak in Lake Volta/ Ghana (2018-2019).

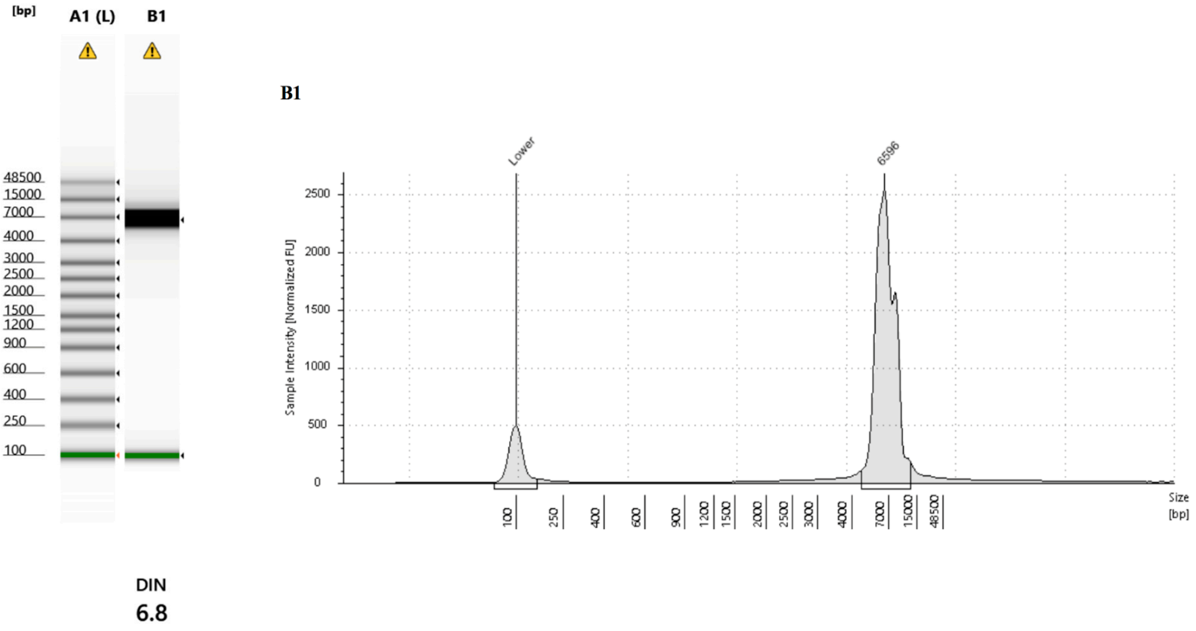

(a) (b)

Figure S3. Gel-like image and electropherogram of the 6kb ISKNV amplicons targeting the full ISKNV genome. (**a**). Amplicons of interest were observed as a thick band at the expected location. (**b**). The x-axis on the electropherogram represents amplicon size (bp), while the y-axis represents the measurement response of fluorescence units (FUs). Highly intact DNA was shown as a narrow peak above the highest marker peak of 6,956 bp.

(A)

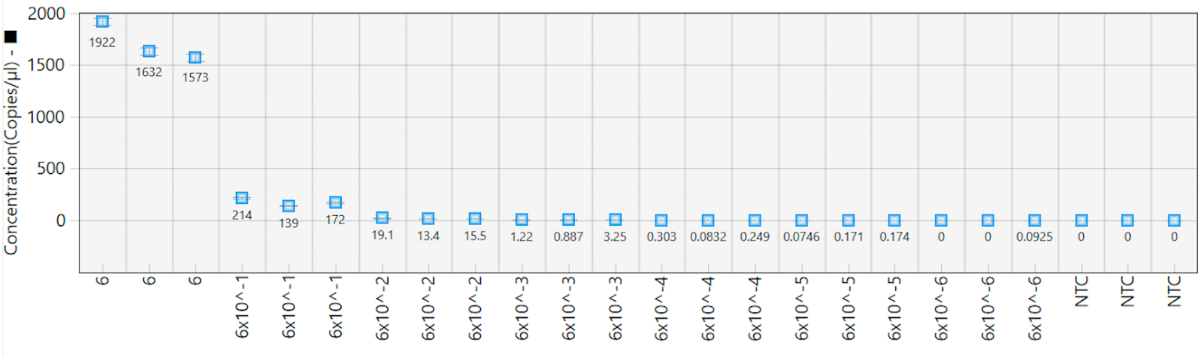

(b)

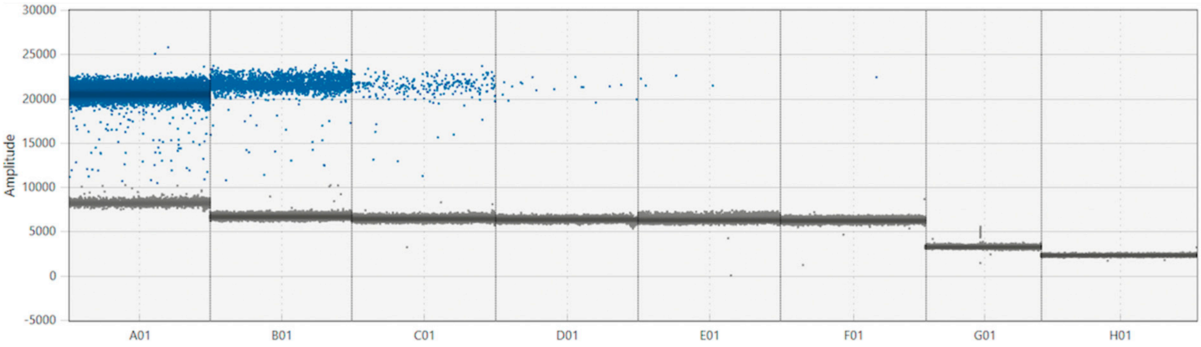

(c)

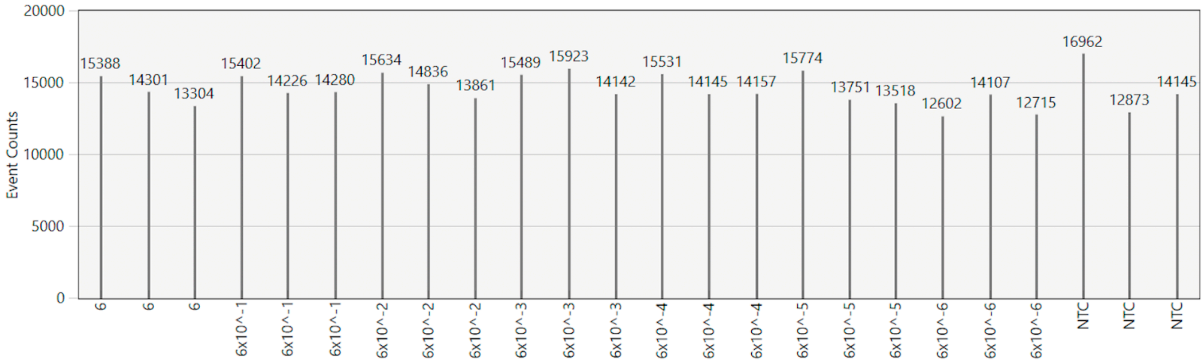

Figure S4. The ddPCR output data showing the number of templates generated for a serial dilution in triplicates of ISKNV. **a.** One-dimensional scatterplot of event number (droplets) vs. fluorescence amplitude for one set of serial dilutions, showing an ideal assay with a clear separation of positive (blue) and negative (grey) droplets **b.** Copies of ISKNV template/µL for all dilutions **c.** Event count of the total number of droplets generated.

Table S1

Different ISKNV PCR primer schemes used by the ZiBRA project generated by the Primal Scheme software [23] using: a. the reference genome (NC_003494). b. the reference genome (NC_003494), with four primer pairs replacing the dropping regions. c. the genome alignment created from the reference genome. d. the reference (NC_003494), targeting 6kb amplicons.

a. V1.

Name	Pool	Sequence	Length
**ShaymaAlath_1_LEFT**	ISKNV_1	AGTGTGCAGAGCATCCATGTTG	22
**ShaymaAlath_1_RIGHT**	ISKNV_1	ACAGTGGTTGTCCGTACCAGAA	22
**ShaymaAlath_2_LEFT**	ISKNV_2	AGCACATCACATATTGTAAAGGCCA	25
**ShaymaAlath_2_RIGHT**	ISKNV_2	CCATGGGTTCAACCAACTACGG	22
**ShaymaAlath_3_LEFT**	ISKNV_1	AAACTTTTGGGCCACCGTGTAG	22
**ShaymaAlath_3_RIGHT**	ISKNV_1	CGTCAAGCCCATGATACGCTAC	22
**ShaymaAlath_4_LEFT**	ISKNV_2	TACCGCTTTCACTGTGCAGGTA	22
**ShaymaAlath_4_RIGHT**	ISKNV_2	CCACACGTCACATAGTTCTGCC	22
**ShaymaAlath_5_LEFT**	ISKNV_1	GACACTGTGTTTATCTGTCGTGGA	24
**ShaymaAlath_5_RIGHT**	ISKNV_1	GGGTGGTGTTGCCCTAATCAAG	22
**ShaymaAlath_6_LEFT**	ISKNV_2	AGCTTGTCGATGTGCTGGTAAC	22
**ShaymaAlath_6_RIGHT**	ISKNV_2	CCCAACCTGTGCACCAAGTATC	22
**ShaymaAlath_7_LEFT**	ISKNV_1	ATGTCAACAGTCATAACGCCCG	22
**ShaymaAlath_7_RIGHT**	ISKNV_1	TTGTCAAACACCAACTTGGCCA	22
**ShaymaAlath_8_LEFT**	ISKNV_2	GTCGGATGCCACAGAGAAGTCT	22

**ShaymaAlath_8_RIGHT**	ISKNV_2	GAAACGCAGGTCACCCACTAAG	22
**ShaymaAlath_9_LEFT**	ISKNV_1	GGACATGTGCGCATCTAACGAC	22
**ShaymaAlath_9_RIGHT**	ISKNV_1	TGCAAGACACCAATCTCGATGC	22
**ShaymaAlath_10_LEFT**	ISKNV_2	CCGAGCATCATCATATCCAAGAACA	25
**ShaymaAlath_10_RIGHT**	ISKNV_2	GTTGCTGTATCCGAACACCTGG	22
**ShaymaAlath_11_LEFT**	ISKNV_1	GGTGATTGGCGTCACTGTATGG	22
**ShaymaAlath_11_RIGHT**	ISKNV_1	ATGTACCACCTCGCCATGTACA	22
**ShaymaAlath_12_LEFT**	ISKNV_2	AATTGACAACCAGACGACCACC	22
**ShaymaAlath_12_RIGHT**	ISKNV_2	TTTGCATTCTCTCTTGGGTGGC	22
**ShaymaAlath_13_LEFT**	ISKNV_1	CACCGTAGCAACCACTACAGTG	22
**ShaymaAlath_13_RIGHT**	ISKNV_1	TGTGTGTTATTAGAAATCTTCAGTCATTGT	30
**ShaymaAlath_14_LEFT**	ISKNV_2	AAACAAACTTCTTTGAACGCCGT	23
**ShaymaAlath_14_RIGHT**	ISKNV_2	TCGCCACCGACTATCTGTAGTG	22
**ShaymaAlath_15_LEFT**	ISKNV_1	GTTGCTGTATGGTAGCCACTGC	22
**ShaymaAlath_15_RIGHT**	ISKNV_1	TGTCGTGGTATCCCTTCAGCAT	22
**ShaymaAlath_16_LEFT**	ISKNV_2	ATCGCGGGCACTTTCCATTAAC	22
**ShaymaAlath_16_RIGHT**	ISKNV_2	GGATATCGGCCGGTTTGTGTTT	22
**ShaymaAlath_17_LEFT**	ISKNV_1	TCTGACGGCAACATAAATGGCC	22
**ShaymaAlath_17_RIGHT**	ISKNV_1	TTTCCATGCAAGGCGACATTGA	22
**ShaymaAlath_18_LEFT**	ISKNV_2	TCGTACAGGCACATCTTCCTCC	22
**ShaymaAlath_18_RIGHT**	ISKNV_2	TTGGTGATGGCATTGACAGAGC	22
**ShaymaAlath_19_LEFT**	ISKNV_1	GCAATCTGTTCAAGCAGTGGGT	22
**ShaymaAlath_19_RIGHT**	ISKNV_1	TTCCCCAATTTTTATGCCCCCG	22
**ShaymaAlath_20_LEFT**	ISKNV_2	AAAGCATCTGGTGGCCAACAAG	22
**ShaymaAlath_20_RIGHT**	ISKNV_2	CGTGTTTGTCATAGGCACCCTC	22
**ShaymaAlath_21_LEFT**	ISKNV_1	GCGCATTGTCACACAGCACATA	22
**ShaymaAlath_21_RIGHT**	ISKNV_1	TATCCTGTAGACAAGGACGCGG	22
**ShaymaAlath_22_LEFT**	ISKNV_2	ACGTGTCATGTCTATAAGCATGCG	24
**ShaymaAlath_22_RIGHT**	ISKNV_2	GCGCATAGCCACAGATACTGTC	22
**ShaymaAlath_23_LEFT**	ISKNV_1	GTACAATCAGCCGTGTGACAGC	22
**ShaymaAlath_23_RIGHT**	ISKNV_1	TGTCTATGTGCACGATGGGTCT	22
**ShaymaAlath_24_LEFT**	ISKNV_2	TTACACGTGGGTCTAGGGACAC	22

**ShaymaAlath_24_RIGHT**	ISKNV_2	TATTGCCAAAACCACGGACGAG	22
**ShaymaAlath_25_LEFT**	ISKNV_1	GACATTTGTGGTGCACGCAGAA	22
**ShaymaAlath_25_RIGHT**	ISKNV_1	CGTGCTATGTATACGCGCATGT	22
**ShaymaAlath_26_LEFT**	ISKNV_2	GTTCCAGAACAAGACACACGGT	22
**ShaymaAlath_26_RIGHT**	ISKNV_2	ACAACACCACTTGCTGTGTACG	22
**ShaymaAlath_27_LEFT**	ISKNV_1	CTGTTCTGGAGACGAGGCTACT	22
**ShaymaAlath_27_RIGHT**	ISKNV_1	GACGCTGACCTGAGTGCTATTG	22
**ShaymaAlath_28_LEFT**	ISKNV_2	AGGAACGGCATTTTAAATTGGGAAG	25
**ShaymaAlath_28_RIGHT**	ISKNV_2	CAGCTGCGCAACAATAGGTACA	22
**ShaymaAlath_29_LEFT**	ISKNV_1	CAACCTGGGCTGCTCACATATG	22
**ShaymaAlath_29_RIGHT**	ISKNV_1	ATGATGACAACTCTTGCGCTGG	22
**ShaymaAlath_30_LEFT**	ISKNV_2	CACTGTTGTTGTGCAGTAGTCAC	23
**ShaymaAlath_30_RIGHT**	ISKNV_2	CGATCGCTATTATGCACCCCAC	22
**ShaymaAlath_31_LEFT**	ISKNV_1	TACGCCTCCAGAACATCGTCAA	22
**ShaymaAlath_31_RIGHT**	ISKNV_1	CGGCAGGTTACATACACACCAC	22
**ShaymaAlath_32_LEFT**	ISKNV_2	ATCCTCAATGGGCAGCTTGGTA	22
**ShaymaAlath_32_RIGHT**	ISKNV_2	GCCGGTGGGATATTATGGCATG	22
**ShaymaAlath_33_LEFT**	ISKNV_1	CATTTGTCCATGTCCACGCACC	22
**ShaymaAlath_33_RIGHT**	ISKNV_1	TCAGGGTGCAAAGAAAGTGCTG	22
**ShaymaAlath_34_LEFT**	ISKNV_2	CATCCGGTGGCAATATGAGGTT	22
**ShaymaAlath_34_RIGHT**	ISKNV_2	ACATACGGCTTCAATCGCACTG	22
**ShaymaAlath_35_LEFT**	ISKNV_1	GTAGTCTGCCTTGTACATGCCG	22
**ShaymaAlath_35_RIGHT**	ISKNV_1	GTACAGGACAGCATTGGGAACG	22
**ShaymaAlath_36_LEFT**	ISKNV_2	CCAAGTTGGGTTATTGTAACCGTCA	25
**ShaymaAlath_36_RIGHT**	ISKNV_2	TGACGACAAGCTATTGGTGCAC	22
**ShaymaAlath_37_LEFT**	ISKNV_1	AACTGTTGTAGCTCGTTGCCTC	22
**ShaymaAlath_37_RIGHT**	ISKNV_1	CCATGCTTGTATCTCATCGGCC	22
**ShaymaAlath_38_LEFT**	ISKNV_2	AAGATGCTGTACTTTGTGGCGC	22
**ShaymaAlath_38_RIGHT**	ISKNV_2	ATGTGCAGCGACATCTCAATGG	22
**ShaymaAlath_39_LEFT**	ISKNV_1	CTGTCTGTATGTCACGAAGGGC	22
**ShaymaAlath_39_RIGHT**	ISKNV_1	TCCTGAAGTTCAAGCATTCGGC	22
**ShaymaAlath_40_LEFT**	ISKNV_2	CCAAAGTGGCGTGTGATGTCAT	22

**ShaymaAlath_40_RIGHT**	ISKNV_2	AGCTACCCAATGTCGTACGTCA	22
**ShaymaAlath_41_LEFT**	ISKNV_1	ACACGGCTTGACATACTGTTCG	22
**ShaymaAlath_41_RIGHT**	ISKNV_1	CCGATACCCCAAACATTACGGC	22
**ShaymaAlath_42_LEFT**	ISKNV_2	GGTATGGCAAGGTCACGTCATC	22
**ShaymaAlath_42_RIGHT**	ISKNV_2	TGGTGAAGAAGGGCCCTATGTT	22
**ShaymaAlath_43_LEFT**	ISKNV_1	CTTTACGCCCACATTCTCGGAG	22
**ShaymaAlath_43_RIGHT**	ISKNV_1	CGTATGCGTGTGTTCCAGACAA	22
**ShaymaAlath_44_LEFT**	ISKNV_2	CGAGACCATCACATTTGTCGGT	22
**ShaymaAlath_44_RIGHT**	ISKNV_2	TCGGTTCACCACGTTGAAATGG	22
**ShaymaAlath_45_LEFT**	ISKNV_1	AAATGCCCATATGCGCCGTTTC	22
**ShaymaAlath_45_RIGHT**	ISKNV_1	ACAATCTAGCTCCAGGTGCTGT	22
**ShaymaAlath_46_LEFT**	ISKNV_2	ATAGTGGGATCTGTGGCACCTG	22
**ShaymaAlath_46_RIGHT**	ISKNV_2	TCCTGGGAAAAGAGTGTCAGGG	22
**ShaymaAlath_47_LEFT**	ISKNV_1	CAGTACCCGCACATACTTGAGC	22
**ShaymaAlath_47_RIGHT**	ISKNV_1	GGCGGTCACATACAACCTTCAG	22
**ShaymaAlath_48_LEFT**	ISKNV_2	TCGTCAGAGTTGGGGTCGTTTA	22
**ShaymaAlath_48_RIGHT**	ISKNV_2	ATTATGCATTGTGCCGTGCTCA	22
**ShaymaAlath_49_LEFT**	ISKNV_1	GACAGCATATGCACCGATGTCG	22
**ShaymaAlath_49_RIGHT**	ISKNV_1	GAAACTACGTGACCAGACGCTG	22
**ShaymaAlath_50_LEFT**	ISKNV_2	GAGCTGTCTACATTGCGCACAA	22
**ShaymaAlath_50_RIGHT**	ISKNV_2	TTGAGCATGCGTATGTGGTGTC	22
**ShaymaAlath_51_LEFT**	ISKNV_1	AGCCGTTGGAGATCATTGTTCT	22
**ShaymaAlath_51_RIGHT**	ISKNV_1	GTTTCCTTCGGCCATCTCCTTG	22
**ShaymaAlath_52_LEFT**	ISKNV_2	TTCTTGTGTGAGGACCCCAAGA	22
**ShaymaAlath_52_RIGHT**	ISKNV_2	TTGGTCTCTGTGGTCATGGGTT	22
**ShaymaAlath_53_LEFT**	ISKNV_1	TGTGTGGTACAATAAACAGTACAAAATACA	30
**ShaymaAlath_53_RIGHT**	ISKNV_1	GTTCAAGGCGTACATGACAGCA	22
**ShaymaAlath_54_LEFT**	ISKNV_2	CATGACGTCAATTAGGTGGCCG	22
**ShaymaAlath_54_RIGHT**	ISKNV_2	ATGGTCGCATGCGTTACAAGAG	22
**ShaymaAlath_55_LEFT**	ISKNV_1	TGGCTGTTGTTGTATCATCAACTGT	25
**ShaymaAlath_55_RIGHT**	ISKNV_1	GTATGTCGGCATTGTCTGTGCA	22
**ShaymaAlath_56_LEFT**	ISKNV_2	GACACACGACACACCTGACAAC	22

**ShaymaAlath_56_RIGHT**	ISKNV_2	TGGGTAGTTGGTTCCCATTCGT	22
**ShaymaAlath_57_LEFT**	ISKNV_1	GGGCATGCTGTCCAACAACATA	22
**ShaymaAlath_57_RIGHT**	ISKNV_1	TGCCTGTACTCACGCCATATCA	22
**ShaymaAlath_58_LEFT**	ISKNV_2	GGGAGGGCTTAACGGAGATGTT	22
**ShaymaAlath_58_RIGHT**	ISKNV_2	GCCGACTGAGCCAATGTGATAG	22
**ShaymaAlath_59_LEFT**	ISKNV_1	GAGATTGGAGATGTACTGGCCG	22
**ShaymaAlath_59_RIGHT**	ISKNV_1	CCAGGAGAACACAAAGGATGGC	22
**ShaymaAlath_60_LEFT**	ISKNV_2	TTGCCTCGAGCTGGTTGACAAA	22
**ShaymaAlath_60_RIGHT**	ISKNV_2	CTCCATGGTGTCTGTTGATGCC	22
**ShaymaAlath_61_LEFT**	ISKNV_1	CATGCTGGTGTCGTAGCGTATG	22
**ShaymaAlath_61_RIGHT**	ISKNV_1	CGTGTGATAATGTCGGCGTCAA	22
**ShaymaAlath_62_LEFT**	ISKNV_2	GGACACAATGACACGACAGGTT	22
**ShaymaAlath_62_RIGHT**	ISKNV_2	GCTGTGATGACAAGAGACCTGC	22

b. **V2.**

**ShaymaAlath_1_LEFT**	ShaymaAlath_1	AGTGTGCAGAGCATCCATGTTG	22
**ShaymaAlath_1_RIGHT**	ShaymaAlath_1	ACAGTGGTTGTCCGTACCAGAA	22
**ShaymaAlath_2_LEFT**	ShaymaAlath_2	AGCACATCACATATTGTAAAGGCCA	25
**ShaymaAlath_2_RIGHT**	ShaymaAlath_2	CCATGGGTTCAACCAACTACGG	22
**ShaymaAlath_3_LEFT**	ShaymaAlath_1	AAACTTTTGGGCCACCGTGTAG	22
**ShaymaAlath_3_RIGHT**	ShaymaAlath_1	CGTCAAGCCCATGATACGCTAC	22
**ShaymaAlath_4_LEFT**	ShaymaAlath_2	TACCGCTTTCACTGTGCAGGTA	22
**ShaymaAlath_4_RIGHT**	ShaymaAlath_2	CCACACGTCACATAGTTCTGCC	22
**ShaymaAlath_5_LEFT**	ShaymaAlath_1	GACACTGTGTTTATCTGTCGTGGA	24
**ShaymaAlath_5_RIGHT**	ShaymaAlath_1	GGGTGGTGTTGCCCTAATCAAG	22
**ShaymaAlath_6_LEFT**	ShaymaAlath_2	AGCTTGTCGATGTGCTGGTAAC	22
**ShaymaAlath_6_RIGHT**	ShaymaAlath_2	CCCAACCTGTGCACCAAGTATC	22
**ShaymaAlath_7_LEFT**	ShaymaAlath_1	ATGTCAACAGTCATAACGCCCG	22
**ShaymaAlath_7_RIGHT**	ShaymaAlath_1	TTGTCAAACACCAACTTGGCCA	22
**ShaymaAlath_8_LEFT**	ShaymaAlath_2	GTCGGATGCCACAGAGAAGTCT	22
**ShaymaAlath_8_RIGHT**	ShaymaAlath_2	GAAACGCAGGTCACCCACTAAG	22
**ShaymaAlath_9_LEFT**	ShaymaAlath_1	GGACATGTGCGCATCTAACGAC	22

**ShaymaAlath_9_RIGHT**	ShaymaAlath_1	TGCAAGACACCAATCTCGATGC	22
**ShaymaAlath_10_LEFT**	ShaymaAlath_2	CCGAGCATCATCATATCCAAGAACA	25
**ShaymaAlath_10_RIGHT**	ShaymaAlath_2	GTTGCTGTATCCGAACACCTGG	22
**ShaymaAlath_11_LEFT**	ShaymaAlath_1	GGTGATTGGCGTCACTGTATGG	22
**ShaymaAlath_11_RIGHT**	ShaymaAlath_1	ATGTACCACCTCGCCATGTACA	22
**ShaymaAlath_12_LEFT**	ShaymaAlath_2	AATTGACAACCAGACGACCACC	22
**ShaymaAlath_12_RIGHT**	ShaymaAlath_2	TTTGCATTCTCTCTTGGGTGGC	22
**ShaymaAlath_13_LEFT**	ShaymaAlath_1	CACCGTAGCAACCACTACAGTG	22
**ShaymaAlath_13_RIGHT**	ShaymaAlath_1	TGTGTGTTATTAGAAATCTTCAGTCATTGT	30
**ShaymaAlath_14_LEFT**	ShaymaAlath_2	AAACAAACTTCTTTGAACGCCGT	23
**ShaymaAlath_14_RIGHT**	ShaymaAlath_2	TCGCCACCGACTATCTGTAGTG	22
**ShaymaAlath_15_LEFT**	ShaymaAlath_1	GTTGCTGTATGGTAGCCACTGC	22
**ShaymaAlath_15_RIGHT**	ShaymaAlath_1	TGTCGTGGTATCCCTTCAGCAT	22
**ShaymaAlath_16_LEFT**	ShaymaAlath_2	ATCGCGGGCACTTTCCATTAAC	22
**ShaymaAlath_16_RIGHT**	ShaymaAlath_2	GGATATCGGCCGGTTTGTGTTT	22
**ShaymaAlath_17_LEFT**	ShaymaAlath_1	TCTGACGGCAACATAAATGGCC	22
**ShaymaAlath_17_RIGHT**	ShaymaAlath_1	TTTCCATGCAAGGCGACATTGA	22
**ShaymaAlath_18_LEFT**	ShaymaAlath_2	TCGTACAGGCACATCTTCCTCC	22
**ShaymaAlath_18_RIGHT**	ShaymaAlath_2	TTGGTGATGGCATTGACAGAGC	22
**ShaymaAlath_19_LEFT**	ShaymaAlath_1	GCAATCTGTTCAAGCAGTGGGT	22
**ShaymaAlath_19_RIGHT**	ShaymaAlath_1	TTCCCCAATTTTTATGCCCCCG	22
**ShaymaAlath_20_LEFT**	ShaymaAlath_2	AAAGCATCTGGTGGCCAACAAG	22
**ShaymaAlath_20_RIGHT**	ShaymaAlath_2	CGTGTTTGTCATAGGCACCCTC	22
**ShaymaAlath_21_LEFT**	ShaymaAlath_1	GCGCATTGTCACACAGCACATA	22
**ShaymaAlath_21_RIGHT**	ShaymaAlath_1	TATCCTGTAGACAAGGACGCGG	22
**ShaymaAlath_22_LEFT**	ShaymaAlath_2	ACGTGTCATGTCTATAAGCATGCG	24
**ShaymaAlath_22_RIGHT**	ShaymaAlath_2	GCGCATAGCCACAGATACTGTC	22
**ISKNV_Alignment_23_LEFT**	ShaymaAlath_1	CTGGTCAACACATCGTCCACAT	22
**ISKNV_Alignment_23_RIGHT**	ShaymaAlath_1	GGGACATGGGCATCGATGTAAA	22
**ShaymaAlath_24_LEFT**	ShaymaAlath_2	TTACACGTGGGTCTAGGGACAC	22
**ShaymaAlath_24_RIGHT**	ShaymaAlath_2	TATTGCCAAAACCACGGACGAG	22
**ShaymaAlath_25_LEFT**	ShaymaAlath_1	GACATTTGTGGTGCACGCAGAA	22

**ShaymaAlath_25_RIGHT**	ShaymaAlath_1	CGTGCTATGTATACGCGCATGT	22
**ShaymaAlath_26_LEFT**	ShaymaAlath_2	GTTCCAGAACAAGACACACGGT	22
**ShaymaAlath_26_RIGHT**	ShaymaAlath_2	ACAACACCACTTGCTGTGTACG	22
**ShaymaAlath_27_LEFT**	ShaymaAlath_1	CTGTTCTGGAGACGAGGCTACT	22
**ShaymaAlath_27_RIGHT**	ShaymaAlath_1	GACGCTGACCTGAGTGCTATTG	22
**ShaymaAlath_28_LEFT**	ShaymaAlath_2	AGGAACGGCATTTTAAATTGGGAAG	25
**ShaymaAlath_28_RIGHT**	ShaymaAlath_1	CAGCTGCGCAACAATAGGTACA	22
**ShaymaAlath_29_LEFT**	ShaymaAlath_2	CAACCTGGGCTGCTCACATATG	22
**ShaymaAlath_29_RIGHT**	ShaymaAlath_2	ATGATGACAACTCTTGCGCTGG	22
**ShaymaAlath_30_LEFT**	ShaymaAlath_1	CACTGTTGTTGTGCAGTAGTCAC	23
**ShaymaAlath_30_RIGHT**	ShaymaAlath_1	CGATCGCTATTATGCACCCCAC	22
**ShaymaAlath_31_LEFT**	ShaymaAlath_2	TACGCCTCCAGAACATCGTCAA	22
**ShaymaAlath_31_RIGHT**	ShaymaAlath_2	CGGCAGGTTACATACACACCAC	22
**ShaymaAlath_32_LEFT**	ShaymaAlath_1	ATCCTCAATGGGCAGCTTGGTA	22
**ShaymaAlath_32_RIGHT**	ShaymaAlath_1	GCCGGTGGGATATTATGGCATG	22
**ShaymaAlath_33_LEFT**	ShaymaAlath_2	CATTTGTCCATGTCCACGCACC	22
**ShaymaAlath_33_RIGHT**	ShaymaAlath_2	TCAGGGTGCAAAGAAAGTGCTG	22
**ShaymaAlath_34_LEFT**	ShaymaAlath_1	CATCCGGTGGCAATATGAGGTT	22
**ShaymaAlath_34_RIGHT**	ShaymaAlath_1	ACATACGGCTTCAATCGCACTG	22
**ShaymaAlath_35_LEFT**	ShaymaAlath_2	GTAGTCTGCCTTGTACATGCCG	22
**ShaymaAlath_35_RIGHT**	ShaymaAlath_2	GTACAGGACAGCATTGGGAACG	22
**ShaymaAlath_36_LEFT**	ShaymaAlath_1	CCAAGTTGGGTTATTGTAACCGTCA	25
**ShaymaAlath_36_RIGHT**	ShaymaAlath_1	TGACGACAAGCTATTGGTGCAC	22
**ShaymaAlath_37_LEFT**	ShaymaAlath_2	AACTGTTGTAGCTCGTTGCCTC	22
**ShaymaAlath_37_RIGHT**	ShaymaAlath_2	CCATGCTTGTATCTCATCGGCC	22
**ShaymaAlath_38_LEFT**	ShaymaAlath_1	AAGATGCTGTACTTTGTGGCGC	22
**ShaymaAlath_38_RIGHT**	ShaymaAlath_1	ATGTGCAGCGACATCTCAATGG	22
**ISKNV_Alignment_39_LEFT**	ShaymaAlath_2	CTGTCTGTATGTCACGAAGGGC	22
**ISKNV_Align-ment_39_RIGHT**	ShaymaAlath_2	TAGCGTGTCCTGAAGTTCAAGC	22
**ShaymaAlath_40_LEFT**	ShaymaAlath_1	CCAAAGTGGCGTGTGATGTCAT	22
**ShaymaAlath_40_RIGHT**	ShaymaAlath_1	AGCTACCCAATGTCGTACGTCA	22
**ShaymaAlath_41_LEFT**	ShaymaAlath_2	ACACGGCTTGACATACTGTTCG	22

**ShaymaAlath_41_RIGHT**	ShaymaAlath_2	CCGATACCCCAAACATTACGGC	22
**ShaymaAlath_42_LEFT**	ShaymaAlath_1	GGTATGGCAAGGTCACGTCATC	22
**ShaymaAlath_42_RIGHT**	ShaymaAlath_1	TGGTGAAGAAGGGCCCTATGTT	22
**ShaymaAlath_43_LEFT**	ShaymaAlath_2	CTTTACGCCCACATTCTCGGAG	22
**ShaymaAlath_43_RIGHT**	ShaymaAlath_2	CGTATGCGTGTGTTCCAGACAA	22
**ShaymaAlath_44_LEFT**	ShaymaAlath_1	CGAGACCATCACATTTGTCGGT	22
**ShaymaAlath_44_RIGHT**	ShaymaAlath_1	TCGGTTCACCACGTTGAAATGG	22
**ShaymaAlath_45_LEFT**	ShaymaAlath_2	AAATGCCCATATGCGCCGTTTC	22
**ShaymaAlath_45_RIGHT**	ShaymaAlath_2	ACAATCTAGCTCCAGGTGCTGT	22
**ShaymaAlath_46_LEFT**	ShaymaAlath_1	ATAGTGGGATCTGTGGCACCTG	22
**ShaymaAlath_46_RIGHT**	ShaymaAlath_1	TCCTGGGAAAAGAGTGTCAGGG	22
**ISKNV_Alignment_47_LEFT**	ShaymaAlath_2	ACAAGACTCGCAGTGTGTTTGA	22
**ISKNV_Align-ment_47_RIGHT**	ShaymaAlath_2	GTAGCATCGTGTCGCGCATAAA	22
**ShaymaAlath_48_LEFT**	ShaymaAlath_1	TCGTCAGAGTTGGGGTCGTTTA	22
**ShaymaAlath_48_RIGHT**	ShaymaAlath_1	ATTATGCATTGTGCCGTGCTCA	22
**ShaymaAlath_49_LEFT**	ShaymaAlath_2	GACAGCATATGCACCGATGTCG	22
**ShaymaAlath_49_RIGHT**	ShaymaAlath_2	GAAACTACGTGACCAGACGCTG	22
**ISKNV_Alignment_50_LEFT**	ShaymaAlath_1	CCATGTGCTTTTTGGCCACATC	22
**ISKNV_Align-ment_50_RIGHT**	ShaymaAlath_1	CGGTTGGGGCATAATACGGAAT	22
**ShaymaAlath_51_LEFT**	ShaymaAlath_2	AGCCCGTTGGAGATCATTGTTCT	22
**ShaymaAlath_51_RIGHT**	ShaymaAlath_2	GTTTCCTTCGGCCATCTCCTTG	22
**ShaymaAlath_52_LEFT**	ShaymaAlath_1	TTCTTGTGTGAGGACCCCAAGA	22
**ShaymaAlath_52_RIGHT**	ShaymaAlath_1	TTGGTCTCTGTGGTCATGGGTT	22
**ShaymaAlath_53_LEFT**	ShaymaAlath_2	TGTGTGGTACAATAAACAG-TACAAAATACA	30
**ShaymaAlath_53_RIGHT**	ShaymaAlath_2	GTTCAAGGCGTACATGACAGCA	22
**ShaymaAlath_54_LEFT**	ShaymaAlath_1	CATGACGTCAATTAGGTGGCCG	22
**ShaymaAlath_54_RIGHT**	ShaymaAlath_1	ATGGTCGCATGCGTTACAAGAG	22
**ShaymaAlath_55_LEFT**	ShaymaAlath_2	TGGCTGTTGTTGTATCATCAACTGT	25
**ShaymaAlath_55_RIGHT**	ShaymaAlath_2	GTATGTCGGCATTGTCTGTGCA	22
**ShaymaAlath_56_LEFT**	ShaymaAlath_1	GACACACGACACACCTGACAAC	22
**ShaymaAlath_56_RIGHT**	ShaymaAlath_1	TGGGTAGTTGGTTCCCATTCGT	22

**ShaymaAlath_57_LEFT**	ShaymaAlath_2	GGGCATGCTGTCCAACAACATA	22
**ShaymaAlath_57_RIGHT**	ShaymaAlath_2	TGCCTGTACTCACGCCATATCA	22
**ShaymaAlath_58_LEFT**	ShaymaAlath_1	GGGAGGGCTTAACGGAGATGTT	22
**ShaymaAlath_58_RIGHT**	ShaymaAlath_1	GCCGACTGAGCCAATGTGATAG	22
**ShaymaAlath_59_LEFT**	ShaymaAlath_2	GAGATTGGAGATGTACTGGCCG	22
**ShaymaAlath_59_RIGHT**	ShaymaAlath_2	CCAGGAGAACACAAAGGATGGC	22
**ShaymaAlath_60_LEFT**	ShaymaAlath_1	TTGCCTCGAGCTGGTTGACAAA	22
**ShaymaAlath_60_RIGHT**	ShaymaAlath_1	CTCCATGGTGTCTGTTGATGCC	22
**ShaymaAlath_61_LEFT**	ShaymaAlath_2	CATGCTGGTGTCGTAGCGTATG	22
**ShaymaAlath_61_RIGHT**	ShaymaAlath_2	CGTGTGATAATGTCGGCGTCAA	22
**ShaymaAlath_62_LEFT**	ShaymaAlath_1	GGACACAATGACACGACAGGTT	22
**ShaymaAlath_62_RIGHT**	ShaymaAlath_1	GCTGTGATGACAAGAGACCTGC	22

c. **V3**

**scheme_1_LEFT**	scheme_1	GGCGCCTGTAATATAGCCATGT	22
**scheme_1_RIGHT**	scheme_1	ACCAACGTTGTCTTGGCGAATA	22
**scheme_2_LEFT**	scheme_2	TGAAAAGGCAGAGCACATCACA	22
**scheme_2_RIGHT**	scheme_2	TGGACATGGGCAATATCAACCC	22
**scheme_3_LEFT**	scheme_1	GGGGCAATCCATAGCTTACAGG	22
**scheme_3_RIGHT**	scheme_1	AAATTCACTGACGTCACCCTGG	22
**scheme_4_LEFT**	scheme_2	GCCAGGTTCAGTTTGTAGCGTA	22
**scheme_4_RIGHT**	scheme_2	GTTTGCCACACACGTGTACTTG	22
**scheme_5_LEFT**	scheme_1	GTAGGTCACATGAAAGGGCCAG	22
**scheme_5_RIGHT**	scheme_1	AGCAGAATGGATGGTTGCATGT	22
**scheme_6_LEFT**	scheme_2	TTCGATGTCTGTCGGCTTGTTG	22
**scheme_6_RIGHT**	scheme_2	GTTAAGGCGACCGAGCTTTACC	22
**scheme_7_LEFT**	scheme_1	CAAATAGCATGTTGCAGCACCG	22
**scheme_7_RIGHT**	scheme_1	CCGGCACTCTTTCCAGTATCTG	22
**scheme_8_LEFT**	scheme_2	AGACGTGCTAAAGCGACATGAC	22
**scheme_8_RIGHT**	scheme_2	GGTGCATTCGACATACACCACT	22
**scheme_9_LEFT**	scheme_1	GCTGGCAGTGCACTTGAAGATA	22
**scheme_9_RIGHT**	scheme_1	CTTAAGGAGATGAGCGGCCTTC	22

**scheme_10_LEFT**	scheme_2	CGACATTAGGGCAGTTCTGGAC	22
**scheme_10_RIGHT**	scheme_2	TCCACACTGTTGGCCCTATACT	22
**scheme_11_LEFT**	scheme_1	CCGGGGTGCTGTATGACATATG	22
**scheme_11_RIGHT**	scheme_1	ATTCGATTGGTAGCACTGGAGC	22
**scheme_12_LEFT**	scheme_2	CGGAATGGACGACTACGATGAC	22
**scheme_12_RIGHT**	scheme_2	TTCATTTTGGATGGCAGGTGGT	22
**scheme_13_LEFT**	scheme_1	TGCAAAAAGAAGACCACCACCA	22
**scheme_13_RIGHT**	scheme_1	TGCATGTCAGGCCTGTGGTA	20
**scheme_14_LEFT**	scheme_2	CGCCAAGCAGCATGGAATTTAC	22
**scheme_14_RIGHT**	scheme_2	TCAGCAAAGCCACATTTGAGGA	22
**scheme_15_LEFT**	scheme_1	CCCATTTTTAACTACGCGCCAC	22
**scheme_15_RIGHT**	scheme_1	CACTCAGTGGGGGTTACGATTC	22
**scheme_16_LEFT**	scheme_2	GCGGGCACTTTCCATTAACATC	22
**scheme_16_RIGHT**	scheme_2	ATAAAGAGGCTGAACAGGACCG	22
**scheme_17_LEFT**	scheme_1	GTACATGCCAAACACAAACCGG	22
**scheme_17_RIGHT**	scheme_1	GACAAAGAAGAGGGCGATTCGT	22
**scheme_18_LEFT**	scheme_2	CATAAGGCAGGGTCATCATGGG	22
**scheme_18_RIGHT**	scheme_2	CGGTGTGTATGTGTTCTTGCTG	22
**scheme_19_LEFT**	scheme_1	ACTGTCAAGCGTGTGATGGAAA	22
**scheme_19_RIGHT**	scheme_1	CGCTCATAGTGGTCATGCTCTC	22
**scheme_20_LEFT**	scheme_2	TAAAGCATCTGGTGGCCAACAA	22
**scheme_20_RIGHT**	scheme_2	TCATAGGCACCCTCCATGTCAT	22
**scheme_21_LEFT**	scheme_1	AAAAGGACCTCGAAGGCAAACT	22
**scheme_21_RIGHT**	scheme_1	AGACATGACACGTGACATGAGT	22
**scheme_22_LEFT**	scheme_2	ACCGCTATATGTCTGCCACAAC	22
**scheme_22_RIGHT**	scheme_2	ACAACATGTAGGCCAGCTGAAG	22
**scheme_23_LEFT**	scheme_1	CTGGTCAACACATCGTCCACAT	22
**scheme_23_RIGHT**	scheme_1	GGGACATGGGCATCGATGTAAA	22
**scheme_24_LEFT**	scheme_2	ACCGCTCACGCATAAGCTTAAT	22
**scheme_24_RIGHT**	scheme_2	ACAACTTCGGCTATCCCTCTCA	22
**scheme_25_LEFT**	scheme_1	AATGGAGTCACAGCTTCTTGCC	22
**scheme_25_RIGHT**	scheme_1	TTCCAACTACACTGTGCTGTGC	22

**scheme_26_LEFT**	scheme_2	CACGTCGAGCCGTCTTATGAAT	22
**scheme_26_RIGHT**	scheme_2	TTGTCGATGCTCTCCTTGACAC	22
**scheme_27_LEFT**	scheme_1	ACGTGACACTGATGGAGAGGAT	22
**scheme_27_RIGHT**	scheme_1	GACCAGTCGACATATGTGCCTC	22
**scheme_28_LEFT**	scheme_2	ATTCGCCAATACGTGATCTGGG	22
**scheme_28_RIGHT**	scheme_2	CACGAATCGCAAAGCACACAAA	22
**scheme_29_LEFT**	scheme_1	ATACAACAGGTCGTCAATGGGC	22
**scheme_29_RIGHT**	scheme_1	AGCACCAACTCGTACAACTGTC	22
**scheme_30_LEFT**	scheme_2	TGGTAACATCCCAGTTGTGCTG	22
**scheme_30_RIGHT**	scheme_2	CTAAAGGTCAGTGACGTGGAGC	22
**scheme_31_LEFT**	scheme_1	TCCCCAAAGTCCCTGATGGTAA	22
**scheme_31_RIGHT**	scheme_1	CAAGACGAGCCAACCTTCAGAC	22
**scheme_32_LEFT**	scheme_2	GCTTGACACGTCTCTCATAGGC	22
**scheme_32_RIGHT**	scheme_2	CAACGCGCACACAATGTCATAC	22
**scheme_33_LEFT**	scheme_1	TAGGTTACGCACGGTGTTTGAA	22
**scheme_33_RIGHT**	scheme_1	ATAGTGGGGTGCGTGTAGTGTA	22
**scheme_34_LEFT**	scheme_2	GATGCCTTGCGTGTACTTGTGA	22
**scheme_34_RIGHT**	scheme_2	GGCATGTACAAGGCAGACTACA	22
**scheme_35_LEFT**	scheme_1	TACAATCTCCGCAGCCAACAC	21
**scheme_35_RIGHT**	scheme_1	CGATGACATCTCTGCAAAACGC	22
**scheme_36_LEFT**	scheme_2	CAATGTACGTGCAAGGACTCCA	22
**scheme_36_RIGHT**	scheme_2	AGGCAACGAGCTACAACAGTTT	22
**scheme_37_LEFT**	scheme_1	GCAACACGTTCAGTAGCCTGTA	22
**scheme_37_RIGHT**	scheme_1	TGTGCACCATGGGATTGTAGTG	22
**scheme_38_LEFT**	scheme_2	TCTGTGTGCCGTCATCTTTGAG	22
**scheme_38_RIGHT**	scheme_2	TCTTCGAGGTATCCGGGATCTG	22
**scheme_39_LEFT**	scheme_1	CTGTCTGTATGTCACGAAGGGC	22
**scheme_39_RIGHT**	scheme_1	TAGCGTGTCCTGAAGTTCAAGC	22
**scheme_40_LEFT**	scheme_2	GAATGCTTGCCGAACGGATGTA	22
**scheme_40_RIGHT**	scheme_2	CCGCCGTACCCAGTGTATATTG	22
**scheme_41_LEFT**	scheme_1	TATGCTGTGCCTCAAAGGTGTC	22
**scheme_41_RIGHT**	scheme_1	TTGTAGATGACGTGACCTTGCC	22

**scheme_42_LEFT**	scheme_2	GCATGTTGTCAGGGTACTTGGT	22
**scheme_42_RIGHT**	scheme_2	GAGAATGTGGGCGTAAAGGTGT	22
**scheme_43_LEFT**	scheme_1	CCATGTCCCACCGTACATGTAC	22
**scheme_43_RIGHT**	scheme_1	ACCGACAAATGTGATGGTCTCG	22
**scheme_44_LEFT**	scheme_2	ATGTGGTTTCTGACACTCAGGC	22
**scheme_44_RIGHT**	scheme_2	ACTGTGCACATCTCACGTACAC	22
**scheme_45_LEFT**	scheme_1	CCCACGAATGTACATGAGGCAT	22
**scheme_45_RIGHT**	scheme_1	CAATCTAGCTCCAGGTGCTGTC	22
**scheme_46_LEFT**	scheme_2	TGTCTTCAAGCCGTCTTTGTGT	22
**scheme_46_RIGHT**	scheme_2	CAAGTATGTGCGGGTACTGTCC	22
**scheme_47_LEFT**	scheme_1	ACAAGACTCGCAGTGTGTTTGA	22
**scheme_47_RIGHT**	scheme_1	GTAGCATCGTGTCGCGCATAAA	22
**scheme_48_LEFT**	scheme_2	GACCTTGTCATACAGCGGATCG	22
**scheme_48_RIGHT**	scheme_2	TTTGGTCGCGTGGACATTATGT	22
**scheme_49_LEFT**	scheme_1	CGCACATCGTCAGTATTGTCCA	22
**scheme_49_RIGHT**	scheme_1	GAGATGGACGACACCATGGAAG	22
**scheme_50_LEFT**	scheme_2	CCATGTGCTTTTTGGCCACATC	22
**scheme_50_RIGHT**	scheme_2	CGGTTGGGGCATAATACGGAAT	22
**scheme_51_LEFT**	scheme_1	GCACTGCTGCTACTGAAGAAGG	22
**scheme_51_RIGHT**	scheme_1	GGGCGTTGATGTCGTAGTTGTA	22
**scheme_52_LEFT**	scheme_2	GGACAAGGATGCCATTGTGACT	22
**scheme_52_RIGHT**	scheme_2	AAACCTGCCGTCATACCATAGC	22
**scheme_53_LEFT**	scheme_1	GCTGCTAATACTGTTGCAACGT	22
**scheme_53_RIGHT**	scheme_1	TTATGCGTACCACCGTCATGAC	22
**scheme_54_LEFT**	scheme_2	AAGAGCTTGCAATGAGCCAAGA	22
**scheme_54_RIGHT**	scheme_2	CCTGGCCAGCATTATGTACAGT	22
**scheme_55_LEFT**	scheme_1	TTGTTCAGCACCTGGTGTATGG	22
**scheme_55_RIGHT**	scheme_1	CATGGTTCTGTCTCCGGTGTTT	22
**scheme_56_LEFT**	scheme_2	CACTGTCACGTGTTTGCTGAAC	22
**scheme_56_RIGHT**	scheme_2	TGACATTTGTCTGCTTGTGGTC	22
**scheme_57_LEFT**	scheme_1	GGTGATCTCCTCCTCGCTATCA	22
**scheme_57_RIGHT**	scheme_1	GGACAAGTACACGCTGCTAGAC	22

**scheme_58_LEFT**	scheme_2	TTGCATTCAGGGTGTCGCTTAA	22
**scheme_58_RIGHT**	scheme_2	CCTGTGTAGGAGCTCCAGTACA	22
**scheme_59_LEFT**	scheme_1	CAACTACTTGAGTTCCCCAGGC	22
**scheme_59_RIGHT**	scheme_1	GGCATCATGTTCACCTGTTGTG	22
**scheme_60_LEFT**	scheme_2	CCGTGTCGTCACTACAGTTTGT	22
**scheme_60_RIGHT**	scheme_2	GCAGTCATTTCATGCGAAGACG	22
**scheme_61_LEFT**	scheme_1	TGTTGGCCATGACATCGTACTG	22
**scheme_61_RIGHT**	scheme_1	CTTCGCATCAAACACCCTGAGA	22
**scheme_62_LEFT**	scheme_2	CGGGGTGTGTTGCATTTGTATG	22
**scheme_62_RIGHT**	scheme_2	ACACATTTATGGGCATGCGACT	22

d. **V1.6.**

**Name**	**Pool**	**Sequence**	**Length**
**ISKNV6kb_1_LEFT**	ISKNV_1	AGTGTGCAGAGCATCCATGTTG	22
**ISKNV6kb_1_RIGHT**	ISKNV_1	CGTCAAGCCCATGATACGCTAC	22
**ISKNV6kb_2_LEFT**	ISKNV_2	TACCGCTTTCACTGTGCAGGTA	22
**ISKNV6kb_2_RIGHT**	ISKNV_2	CCCAACCTGTGCACCAAGTATC	22
**ISKNV6kb_3_LEFT**	ISKNV_1	ATGTCAACAGTCATAACGCCCG	22
**ISKNV6kb_3_RIGHT**	ISKNV_1	TGCAAGACACCAATCTCGATGC	22
**ISKNV6kb_4_LEFT**	ISKNV_2	CCGAGCATCATCATATCCAAGAACA	25
**ISKNV6kb_4_RIGHT**	ISKNV_2	TTTGCATTCTCTCTTGGGTGGC	22
**ISKNV6kb_5_LEFT**	ISKNV_1	CACCGTAGCAACCACTACAGTG	22
**ISKNV6kb_5_RIGHT**	ISKNV_1	TGTCGTGGTATCCCTTCAGCAT	22
**ISKNV6kb_6_LEFT**	ISKNV_2	ATCGCGGGCACTTTCCATTAAC	22
**ISKNV6kb_6_RIGHT**	ISKNV_2	TTGGTGATGGCATTGACAGAGC	22
**ISKNV6kb_7_LEFT**	ISKNV_1	GCAATCTGTTCAAGCAGTGGGT	22
**ISKNV6kb_7_RIGHT**	ISKNV_1	TATCCTGTAGACAAGGACGCGG	22
**ISKNV6kb_8_LEFT**	ISKNV_2	ACGTGTCATGTCTATAAGCATGCG	24
**ISKNV6kb_8_RIGHT**	ISKNV_2	TATTGCCAAAACCACGGACGAG	22
**ISKNV6kb_9_LEFT**	ISKNV_1	GACATTTGTGGTGCACGCAGAA	22
**ISKNV6kb_9_RIGHT**	ISKNV_1	GACGCTGACCTGAGTGCTATTG	22

**ISKNV6kb_10_LEFT**	ISKNV_2	AGGAACGGCATTTTAAATTGGGAAG	25
**ISKNV6kb_10_RIGHT**	ISKNV_2	CGATCGCTATTATGCACCCCAC	22
**ISKNV6kb_11_LEFT**	ISKNV_1	TACGCCTCCAGAACATCGTCAA	22
**ISKNV6kb_11_RIGHT**	ISKNV_1	TCAGGGTGCAAAGAAAGTGCTG	22
**ISKNV6kb_12_LEFT**	ISKNV_2	CATCCGGTGGCAATATGAGGTT	22
**ISKNV6kb_12_RIGHT**	ISKNV_2	TGACGACAAGCTATTGGTGCAC	22
**ISKNV6kb_13_LEFT**	ISKNV_1	AACTGTTGTAGCTCGTTGCCTC	22
**ISKNV6kb_13_RIGHT**	ISKNV_1	TCCTGAAGTTCAAGCATTCGGC	22
**ISKNV6kb_14_LEFT**	ISKNV_2	CCAAAGTGGCGTGTGATGTCAT	22
**ISKNV6kb_14_RIGHT**	ISKNV_2	TGGTGAAGAAGGGCCCTATGTT	22
**ISKNV6kb_15_LEFT**	ISKNV_1	CTTTACGCCCACATTCTCGGAG	22
**ISKNV6kb_15_RIGHT**	ISKNV_1	ACAATCTAGCTCCAGGTGCTGT	22
**ISKNV6kb_16_LEFT**	ISKNV_2	ATAGTGGGATCTGTGGCACCTG	22
**ISKNV6kb_16_RIGHT**	ISKNV_2	ATTATGCATTGTGCCGTGCTCA	22
**ISKNV6kb_17_LEFT**	ISKNV_1	GACAGCATATGCACCGATGTCG	22
**ISKNV6kb_17_RIGHT**	ISKNV_1	GTTTCCTTCGGCCATCTCCTTG	22
**ISKNV6kb_18_LEFT**	ISKNV_2	TTCTTGTGTGAGGACCCCAAGA	22
**ISKNV6kb_18_RIGHT**	ISKNV_2	ATGGTCGCATGCGTTACAAGAG	22
**ISKNV6kb_19_LEFT**	ISKNV_1	TGGCTGTTGTTGTATCATCAACTGT	25
**ISKNV6kb_19_RIGHT**	ISKNV_1	TGCCTGTACTCACGCCATATCA	22
**ISKNV6kb_20_LEFT**	ISKNV_2	GGGAGGGCTTAACGGAGATGTT	22
**ISKNV6kb_20_RIGHT**	ISKNV_2	CTCCATGGTGTCTGTTGATGCC	22
**ISKNV6kb_21_LEFT**	ISKNV_2	TTGCCTCGAGCTGGTTGACAAA	22
**ISKNV6kb_21_RIGHT**	ISKNV_2	GCTGTGATGACAAGAGACCTGC	22

Table S2. A list of ISKNV genomes reported in the GenBank and their hosts.

Accession no.	Host	Country	Date	Reference
NC_003494	*Siniperca chuatsi*	China	2001	He et al. 2001 [14]
MT128666	*Lates calcarifer*	Thailand	2018	Kerddee et al. 2021 [44]
MT128667	*Lates calcarifer*	Thailand	2018	Kerddee et al. 2021 [44]
MW273353	*Epalzeorhynchos frenatum*	USA	2018-2019	Koda et al. 2021 [45]
MW273354	*Epalzeorhynchos frenatum*	USA	2018-2019	Koda et al. 2021 [45]

MW46172	*Epinephelus spp.*	Indonesia	2016	Fusianto et al. 2021 [46]
MW557381	*Epinephelus spp.*	Indonesia	2016	Fusianto et al. 2021 [46]
ON212400.1	*Oreochromis nilocticus*	Brazil	2020	Figueiredo et al 2022 [47]

Table S3. Sequencing results for each sequenced sample, collected during the ISKNV outbreak from Lake Volta/ Ghana**.** We show location and date of sampling, the number of sequenced reads, and the percentage of the coverage of MinION reads.

Sample	Farm	Date	No. of reads	% Coverage x20
Farm1.1	1	18.10.2018	23310	89.72
Farm1.2	1	18.10.2018	114824	46.9117
Farm1.3	1	18.10.2018	76204	92.95
Farm1.4	1	18.10.2018	144891	84.99
Farm1.5	1	18.10.2018	146888	86.78
Farm 2.1	2	28.11.2019	24763	84.99
Farm 2.2	2	28.11.2018	11382	44
Farm 2.3	2	28.11.2018	241475	75.38
Farm 2.6	2	28.11.2018	174175	49.96
Farm 2.7	2	28.11.2018	67367	86.54
Farm 2.8	2	28.11.2018	127231	89.59
Farm 2.9	2	28.11.2018	66533	74.63
Farm 2.10	2	28.11.2018	47804	91.39
Farm 2.11	2	28.11.2018	5473	35.22
Farm 2.12	2	28.11.2018	18600	51.18
Farm 2.13	2	28.11.2018	52392	86.22
Farm 2.14	2	28.11.2018	86402	85.04
Farm 6.1	6	10.07.2019	101587	91.72
Farm 6.2	6	10.07.2019	100300	95.91
Farm 6.3	6	10.07.2019	41219	88.53
Farm 6.4	6	10.07.2019	73099	94.42
Farm6.5	6	10.07.2019	538869	94.50
Farm6.6	6	10.07.2019	35121	84.78
Farm6.7	6	10.07.2019	271267	92.71
Farm6.8	6	10.07.2019	435008	89.85
Farm6.9	6	10.07.2019	185147	72.23

Farm6.10	6	10.07.2019	60290	91.39
Farm7.4	7	10.07.2019	44084	89.4
Farm7.1	7	11.07.2019	22914	87.83
Farm7.2	7	11.07.2019	148211	88.23
Farm7.3	7	11.07.2019	3113	44.72
Farm7.5	7	11.07.2019	338190	82.27
Farm 2.1/22	2	20.05.2022	114551	91.16
Farm 2.2/22	2	20.05.2022	177855	89.75
Farm 2.3/22	2	20.05.2022	98879	88.29
Farm 2.4/22	2	20.05.2022	94272	63.48
Farm2.5/22	2	20.05.2022	16934	88.73
Negative cont.	-	20.05.2022	8	0
